# Expression of fibrillin-1 in focal nodular hyperplasia of the liver: a role in microcirculation adaptability

**DOI:** 10.1186/1476-5926-2-S1-S57

**Published:** 2004-01-14

**Authors:** Sébastien Lepreux, Alexis Desmouliere, Jean Rosenbaum, Charles Balabaud, Paulette Bioulac-Sage

**Affiliations:** 1 GREF, INSERM E0362, Université Victor Segalen Bordeaux 2, France; 2 Service d'Anatomie Pathologique, Hépital Pellegrin, C.H.U. Bordeaux, France; 3 Service d'Hépatologie-Gastroentérologie, Hépital Saint-André, C.H.U. Bordeaux, France

## Abstract

**Introduction:**

It has been suggested that the elastic network plays an important role in the tissue response to mechanical stress. The components of the elastic network have been poorly studied in liver diseases. Therefore, in this work, the expression and distribution of fibrillin-1 and elastin were studied in hepatic focal nodular hyperplasia and compared with surrounding liver and hepatocellular adenoma.

**Methods:**

Immunohistochemical studies for fibrillin-1 and elastin were performed on unfixed cryostat sections of focal nodular hyperplasia (22 cases), hepatocellular adenoma (15 cases) and surrounding liver (34 cases).

**Results:**

Surrounding normal liver showed only a continuous, thin and regular immunostaining of fibrillin-1 in the space of Disse, whereas elastin was nearly absent. In focal nodular hyperplasia, fibrillin-1 was more strongly expressed in the perisinusoidal space, compared with surrounding liver; in contrast, in adenomas fibrillin-1 immunostaining was irregular and very low in perisinusoidal space, more intense in peliotic areas.

**Conclusions:**

In focal nodular hyperplasia, the increased microfibrillar network containing fibrillin-1 in the space of Disse could reflect an adaptation of the sinusoidal wall to an increased arterial blood flow in sinusoids. In hepatocellular adenoma, the different patterns of fibrillin-1 could be related to the heterogeneity of the arterial vascularization and to the frequent necrotico-hemorrhagic changes. This study comparing the elastic network in two types of lesions with vascularization abnormalities and in the surrounding liver provides interesting new data for understanding the structural role of fibrillin-1 in the space of Disse.

## Introduction

The elastic fibers in the extra-cellular matrix are composed of elastin associated with 10 nm microfibrils. It has been suggested that the microfibrils mediate the conversion of tropoelastin to elastin and act as a scaffold [1]. However, microfibrils can be present alone [2] and in these situations, their role is not clearly known. Fibrillin-1, a 350 kDa glycoprotein, is a major component of these 10 nm microfibrils [2]. Fibrillin-1 participates in the attachment of cells to the extra-cellular matrix by virtue of RGD motifs [2,3]. Fibrillin-1 and elastin have been widely studied in many human tissues, particularly in the skin and eyes [2,4] but little is known in the liver. We recently studied for the first time, the expression and cellular synthesis of fibrillin-1 in human liver parenchyma [5]. Briefly, i/ in the portal tract, fibrillin-1 was colocalized with elastin in the vessel walls and in connective tissue, whereas a condensation of fibrillin-1 alone was detected close to the basement membrane of bile ducts as well as at the interface with the limiting hepatocytic plates; ii/ fibrillin-1 and elastin were also colocalized in the walls of hepatic vein; iii/ in the liver lobule, fibrillin-1 was detected alone as a regular thin network in the space of Disse all along sinusoids. Although little is known concerning the physiological functions of fibrillin-1 in the liver, it can be assumed that this elastic glycoprotein which is expressed alone in sinusoids, could play a role in the sinusoidal blood flow regulation, in normal, as well as in pathological conditions particularly those exhibiting abnormalities of vascularization.

The aims of our study were:

1. to study the expression and distribution of fibrillin-1 in focal nodular hyperplasia (FNH) which is a pseudo-tumoral benign hepatocytic lesion with a nodular organization, related to an increase in arterial blood flow;

2. to compare these observations with those obtained: a- in a true benign hepatocytic tumor, the hepatocellular adenoma, which is another condition of localized arteriohepatic hyperperfusion, but different, from a physiopathological viewpoint from focal nodular hyperplasia; and b- in the surrounding normal liver.

## Methods

### Human liver specimens

Seventy-one liver samples were studied, obtained from 34 patients. They corresponded to surgically resected specimens of FNH (n = 22), hepatocellular adenoma (n = 15) and the surrounding non-tumoral liver (n = 34). In 3 cases, samples of FNH and hepatocellular adenoma were obtained from the same patient.

One part of tissue samples was routinely formalin fixed and paraffin embedded; five micrometer-thick paraffin sections of each sample were taken and stained with HES for diagnostic purposes. Additional sections were stained with Masson's trichrome, reticulin and orcein. Another part of fresh tissue was immediately snap-frozen in liquid nitrogen-cooled isopentane and stored at -80 degrees C until used for this study. Five micrometer-thick serial frozen sections of each sample were air-dried on Super frost/plus slides (Menzel Glaser, Germany) and processed for immunohistochemistry.

### Immunohistochemistry

Two components of elastic fibers, fibrillin-1 and elastin, as well as alpha-smooth muscle actin (alpha-SMA) for staining of the myofibroblasts (including hepatic stellate cells in their myofibroblastic state) were tested. Mouse monoclonal antibodies against fibrillin-1 (clone 11C1.3; NeoMarkers, Fremont, CA) and against alpha-SMA (Dako A/S, Glostrup, Denmark), and a rabbit polyclonal antibody against human elastin (gift of D. Hartmann, Novotec, Lyon, France) were used. Unfixed frozen sections were incubated with the antibodies against fibrillin-1 (1/100), elastin (1/1000) or alpha-SMA (1/400) diluted in phosphate-buffered saline, pH 7.4 containing 4% bovine serum albumin. After washing, the epitopes were detected with the Envision+ system HRP detection and developed with liquid diaminobenzidine (Dako A/S, Glostrup, Denmark). Sections were examined with a Zeiss Axioplan 2 microscope (Carl Zeiss Microscopy, Jena, Germany). Images were acquired with an AxioCam camera (Carl Zeiss Vision, Hallbergmoos, Germany) by means of the AxioVision image processing and analysis system (Carl Zeiss Vision).

The same immunohistochemical observations were obtained with another anti-fibrillin-1 antibody (clone 69; Chemicon, Temecula, CA) (data not shown). All controls where primary antibodies were omitted were negative.

## Results

### Patients

All main clinical and pathological data are summarized in table 1. All patients with FNH were female, with a mean age of 36.4 years (15é54 years). The mean size of hepatic tumors was 6.45 cm (1é13 cm). Patients with hepatocellular adenoma were predominantly female (12/15) with a mean age of 38.6 years (14é52 years); hepatic tumors were of 7 cm mean size (2.7é13 cm), multiple in 2 cases (cases 27, 33) and/or associated with FNH in 3 cases (cases 20, 21, 22). All female patients were treated with oral contraceptive steroids. One male patient presented alcohol addiction and obesity (case 24), and one was treated with androgenic steroids for a Fanconi disease (case 28).

**Table 1:** Relevant clinical and pathological data

Patient	Age/Sex (years)	Tumor		Non-tumoral Liver
			
		Pathology	Size (cm)	
1	30/F	FNH	5	N
2	15/F	FNH	7.6	N
3	37/F	FNH	2.5	N
4	36/F	FNH	8	N
5	34/F	FNH	7	N
6	41/F	FNH	5.3	N
7	54/F	FNH	7	N
8	14/F	FNH	6	N
9	44/F	FNH	10	N
10	32/F	FNH	7	N
11	35/F	FNH	5.5	N
12	48/F	FNH	8	N
13	32/F	FNH	8	N
14	38/F	FNH	7	Steatosis é Peliosis
15	38/F	FNH	11	N
16	50/F	FNH	2.5	Steatosis
17	44/F	FNH T	7	N
18	39/F	FNH T	3	N
19	34/F	FNH T	8	Steatosis
20	40/F	FNH HCA	2.5 3	Peliosis
21	28/F	FNH HCA	1 4	N
22	38/F	FNH HCA	13 4	N
23	28/F	HCA	8	Steatosis
24	43/M	HCA	3.5	Steatosis
25	43/F	HCA	13	N
26	34/F	HCA	6	N
27	45/F	HCA	10	Peliosis
28	14/M	HCA	2.7	N
29	50/F	HCA	11	Peliosis
30	39/F	HCA	7	N
31	30/F	HCA	10	N
32	49/M	HCA	-	N
33	52/F	HCA	5	N
34	46/F	HCA	11	N

FNH: Focal nodular hyperplasia, FNH T: Telangiectatic focal nodular hyperplasia, HCA: Hepatocellular adenoma, N: Normal

### Histopathology and immunohistochemical analysis

#### Surrounding non tumoral liver tissue

**Figure 1:**
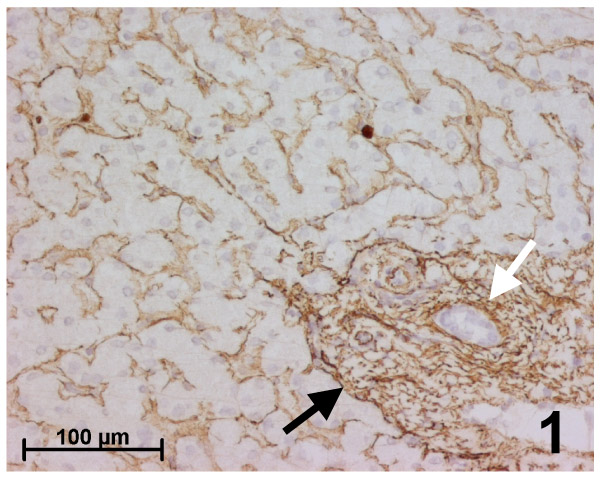
Immunohistochemistry for fibrillin-1 in the surrounding normal liver. In the lobule, fibrillin-1 is detected as a thin, regular network in the space of Disse all along sinusoids. In the portal tract, fibrillin-1 is detected in the vessel walls, in interstitial tissue, with an increase close to the basement membrane of biliary ducts (white arrow) and at the interface with the hepatocytic limiting plate (black arrow).

The surrounding non-tumoral liver tissue (34 cases) showed a normal architecture and cytology. Five cases showed mild steatotic changes (&lt;5%) in parenchyma surrounding hepatocellular adenoma (2 cases) or FNH (3 cases) and there were focal peliotic changes around adenoma (3 cases) or FNH (1 case). Elastic fibers in walls of portal and centrolobular vessels were clearly stained with orcein and no staining was observed in lobular zones. As previously described [5] (Figure 1), fibrillin-1 and elastin were both immunostained in the vessel walls and interstitial tissue in portal tracts; only fibrillin-1 was expressed, while elastin was absent close to the basement membrane of biliary ducts and at the interface with the hepatocytic limiting plate. Both fibrillin-1 and elastin were also present in the wall of centrolobular veins. In the lobules, only fibrillin-1 was detected as a thin, regular network in the space of Disse along sinusoids. There was marked alpha-SMA immunostaining in the vessel walls and in some interstitial cells of the portal tracts and only mild immunostaining in a few hepatic stellate cells of the parenchyma.

#### Focal nodular hyperplasia

Nineteen cases were histologically classified as a classical solid form of FNH, of which one exhibited steatosis (case 21), and 3 cases were telangiectatic FNH (cases 17, 18, 19).

All classical forms of FNH were similar and typical: they displayed numerous nodules of liver parenchyma separated by fibrous septa. Nodules were composed of nearly normal hepatocytes, with large areas of macrovesicular steatosis (>30%) in 1 case (case 21). A fibrous scar was observed in 16 cases and was inconspicuous in 3 cases. The fibrous septa between nodules contained thick-walled arteries with intimal thickening, sometimes partly thrombosed, veins, capillaries and numerous biliary ductules.

Orcein staining clearly showed the elastic lamina of arterial walls, which were sometimes disrupted, the delicate network of elastic fibers in the vein walls and some elastic fibers dispersed in the connective tissue of the septa; elastic fibers were however absent around capillaries. Sinusoids in parenchymal nodules were not stained with orcein.

**Figure 2:**
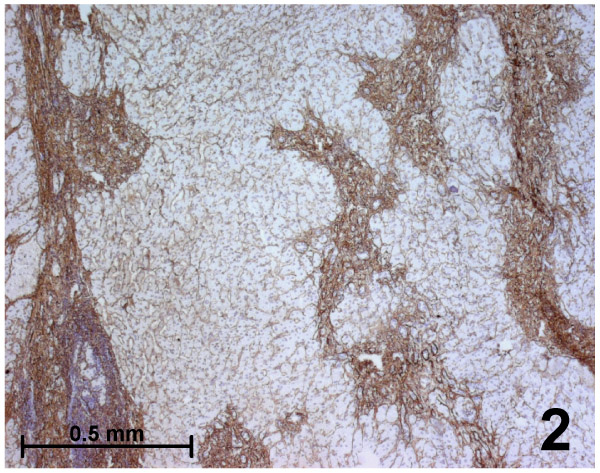
Immunohistochemistry for fibrillin-1 in a classical form of focal nodular hyperplasia. Fibrillin-1 is expressed in fibrous septa separating nodules of liver parenchyma and in between hepatocytic plates.

**Figure 3:**
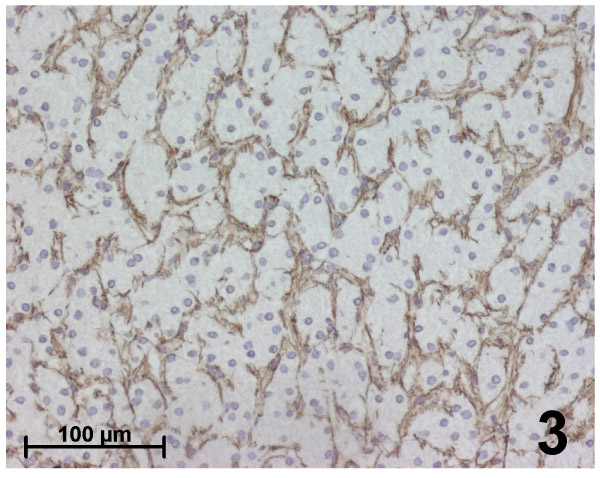
Immunohistochemistry for fibrillin-1 in focal nodular hyperplasia. Within parenchymal nodules, fibrillin-1 is expressed in the perisinusoidal spaces between hepatocytic plates showing a strong, continuous and regular staining.

**Figure 4:**
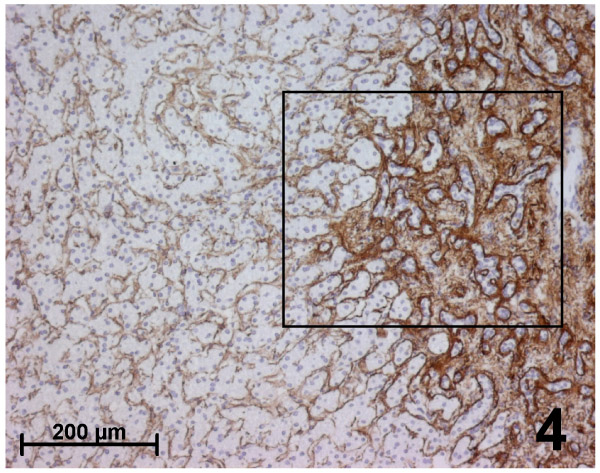
Immunohistochemistry for fibrillin-1 in focal nodular hyperplasia. Fibrillin-1 is expressed in the fibrous septa (right).

**Figure 5:**
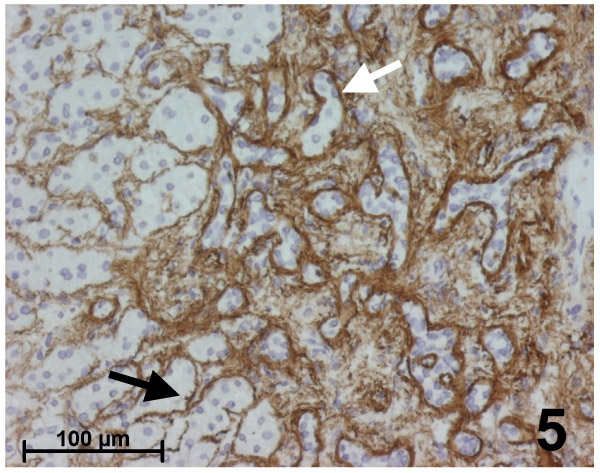
Immunohistochemistry for fibrillin-1 in focal nodular hyperplasia. Higher magnification of the area defined by a rectangle in the Figure 4. Fibrillin-1 staining is stronger close to the basement membrane of biliary ductules (white arrow) in the fibrous septa and around sinusoids near the septa (black arrow).

Immunohistochemical results were identical in all typical FNH (Figures 2,3,4,5):

i. Fibrillin-1 and elastin were both immunostained in the walls of arteries and veins, as well as in the fibrous tissue of septa and fibrous scars (Figures 2, 4, 5).

ii. Fibrillin-1 was expressed alone and more strongly close to the basement membrane of biliary ductules highlighting clearly the characteristic ductular reaction of FNH (Figures 4,5). There was no increase of the fibrillin-1 staining at the interface between the fibrous septa and hepatocytic nodules, as observed between portal tracts and the first hepatocytic plate in normal surrounding liver.

iii. Within parenchymal nodules, only fibrillin-1 was expressed in the perisinusoidal spaces between hepatocytic plates showing strong, continuous and regular staining, with a higher intensity compared with the surrounding normal liver (Figures 3, 4). Furthermore, there was an increase in the staining around sinusoids close to the septa. Elastin staining was sometimes slightly expressed inside hepatocytic nodules, but only close to the fibrous septa.

There was a marked alpha-SMA immunostaining in the artery and vein walls and in some mesenchymal cells of the septa, as well as mild but regular immunostaining of the hepatic stellate cells within the parenchymal nodules, with an increase close to the septa.

The 3 cases of telangiectatic FNH exhibited similar features. They did not display nodules or fibrous scar. However, a few short fibrous septa were found and showed numerous thick-walled arteries, a few veins and a mild degree or no bile ductular reaction. The hepatocytic plates were clearly separated by dilated sinusoids, sometimes alternating with areas of marked ectasia forming occasionally peliotic areas. Orcein stained the elastic lamina of arterial walls and the delicate network of elastic fibers in the vein walls.

Immunohistochemical results were nearly the same as in the classical form. Fibrillin-1 was expressed alone, without elastin in the perisinusoidal spaces between hepatocytic plates, showing a relatively continuous and regular staining, but more accentuated around ectatic sinusoids. There was strong alpha-SMA immunostaining in the vessel walls and moderate but regular immunoreactivity in the hepatic stellate cells of the parenchymal zones, predominantly around dilated sinusoids.

#### Hepatocellular adenoma

**Figure 6:**
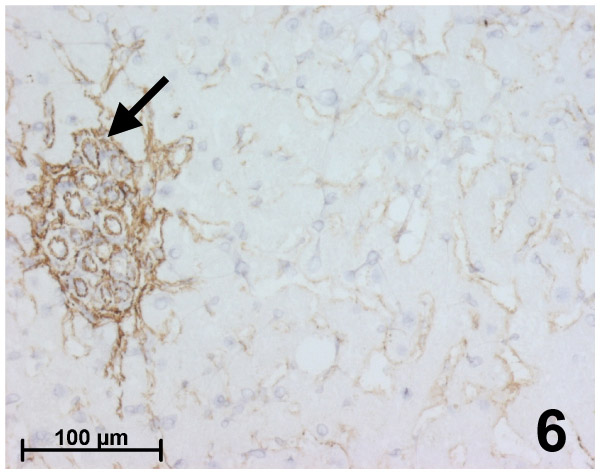
Immunohistochemistry for fibrillin-1 in hepatocellular adenoma. Fibrillin-1 is detected in the numerous arterial sections (black arrow), whereas there is only a weak, discontinuous and irregular staining in the perisinusoidal spaces.

All 15 cases of hepatocellular adenoma were typical. They displayed a loss of the normal lobular architecture without true portal tract and biliary ducts were always absent. The tumoral hepatocyte trabeculae interspersed with thin-walled vessels. Peliotic changes (10 cases), some fibrotic changes (5 cases) adjacent to large areas of necrosis and hemorrhage or massive steatosis (6 cases) were observed. Orcein staining showed the elastic lamina of vessel walls and sometimes a delicate network in the fibrotic changes. Fibrillin-1 and elastin were both immunostained in the vessel walls, especially in the numerous arteries (Figure 6). Within parenchymal zones, fibrillin-1 was sometimes irregularly expressed in the perisinusoidal spaces, showing a discontinuous and irregular staining with lower intensity (8 cases) or with the same intensity (7 cases) compared with the surrounding liver (Figure 6), except in some areas of peliosis where the staining was more strong and continuous. It should be mentioned that, within the same case, there was often heterogeneity of fibrillin-1 staining in perisinusoidal spaces, with alternating stained and unstained zones. Elastin staining was always absent between tumoral hepatocytes. There was marked alpha-SMA cellular immunostaining in the vessel walls and fine and irregular immunostaining in some hepatic stellate cells inside the tumor, which were only clearly stained in the peliotic areas. When present, areas of fibrotic change exhibited strong alpha-SMA cellular immunostaining and strong staining for fibrillin-1 and elastin.

## Discussion

In normal liver, immunohistochemistry is an effective tool for detection of the fibrillin-1 network around sinusoids, since this glycoprotein is expressed alone in space of Disse, as a thin network, without elastin [5] and thus not stained with classical staining of elastic tissue such as orcein. The role of fibrillin-1, a component of elastic tissue, in space of Disse which is a strategic place for exchanges between hepatocytes and sinusoidal microcirculation, remains however to be elucidated. The different patterns of fibrillin-1 expression in FNH and hepatocellular adenoma, in comparison with normal liver, could be a reflection of the response.

In normal liver, sinusoids are supplied by portal vein blood which flows at a low pressure and by hepatic artery, representing respectively two thirds and one third of the total sinusoidal microcirculation [6].

It is well known that FNH are related to an increase in arterial flow due to primary or secondary arterial abnormalities [7-10]. In these conditions, sinusoidal blood flow is modified by an arteriohepatic hyperperfusion, as seen with radiologic imaging and illustrated after gelatin injection [9-12]. It has been reported by Wanless [7,8] that in FNH, sinusoids were directly irrigated with inlet arterioles coming from fibrous septa. Injection of colored gelatin [9] indicated that the abnormal arteries were draining indirectly into sinusoids via capillaries in the fibrous septa. It could be postulated that these vascular conditions lead to an increase in sinusoidal blood pressure and could be an explanation for changing of the sinusoidal endothelial cell phenotype with neo-expression of CD34 and CD31 [9,13] and expression of alpha-SMA in hepatic stellate cells [14]. It is also well known that hepatic stellate cells are active in the adaptation to the changes in sinusoidal blood flow [15].

In comparison with normal surrounding liver, we showed in FNH, in addition to alpha-SMA positive hepatic stellate cells, increased immunostaining for fibrillin-1 in space of Disse, as a continuous and regular network with an enhancement near the fibrous septa. It is interesting to note that sinusoids adjacent to fibrous septa always express vascular endothelial markers such as CD34 [13,14,16].

As FNH represents a condition of arteriohepatic hyperperfusion, it could be thought that the increased arterial blood flow may be a (causal) factor inducing the increase of fibrillin-1 microfibrillar network in space of Disse, as well as phenotypic changes with alpha-SMA neo-expression in cells which secrete fibrillin-1 [5]. As elastic tissue is an important element in the regulation of the blood pressure in vessels, it can be assumed that the increased sinusoidal fibrillin-1 network, which is a component of elastic tissue, is involved in blood pressure regulation along the sinusoidal microvasculature between the hyperplastic hepatocytic plates of FNH.

In telangiectatic FNH which lack the classical signs of FNH but exhibit characteristic features of sinusoidal dilatation, increase of fibrillin-1 network was more irregular, predominating in zones of sinusoidal dilatation. This feature could reflect a predominant increase of arterial perfusion in these dilated areas.

In hepatocellular adenoma, another benign condition of hepatocytic proliferation associated with an irregular and abnormal vascularization, both elastin and fibrillin-1 immunostaining pointed to the great number of vascular sections distributed inside the tumor. The presence of fibrillin-1 alone along sinusoids was discontinuous, irregular and some areas were completely unstained; on the contrary, peliotic zones showed a more intense staining than the non-peliotic ones, but this staining was irregularly distributed within the tumor. Unlike FNH where the microfibrillar organization was correlated with the increase in blood pressure, the lack of a regular and continuous network of microfibrils in the perisinusoidal space in hepatocellular adenoma could explain, at least in part, the frequency of peliosis and necrotico-hemorrhagic complications, characteristic of this tumor. Furthermore, the expression of alpha-SMA in hepatic stellate cells was more inconsistent, underlining again the heterogeneity of the lesion.

## Conclusions

Based on our results, it can be hypothesized that in FNH, the increased microfibrillar network containing fibrillin-1 in the space of Disse could reflect an adaptation of the sinusoidal wall to variations of blood pressure related to an increased arterial blood flow in sinusoids. The different patterns of fibrillin-1 in hepatocellular adenoma could account for the heterogeneity of the arterial vascularization in this tumor characterized by frequent necrotico-hemorrhagic changes. These results could contribute to the understanding of the structural role of fibrillin-1 in space of Disse. Further studies are necessary to affirm whether the changes in fibrillin-1 network are the direct consequence of the liver microvascularisation abnormalities, and in particular of the increased arterial flow in FNH.
